# Patients acceptance and comprehension to written and verbal consent (PAC–VC)

**DOI:** 10.1186/s12910-023-00893-1

**Published:** 2023-02-23

**Authors:** Rabia Kashur, Justin Ezekowitz, Shane Kimber, Robert C. Welsh

**Affiliations:** Division of Cardiology, University of Alberta and Mazankowski Alberta Heart Institute, 2C2 WMC, 8440 – 112 Street NW, Edmonton, AB T6G 2B7 Canada

**Keywords:** Informed consent, Acute myocardial infarction, Verbal assent, Written consent, Clinical trials

## Abstract

**Background:**

Acute myocardial infarction (AMI) research is challenging as it requires enrollment of acutely ill patients. Patients are generally in a suboptimal state for providing informed consent. Patients’ understanding to verbal assents have not been previously examined in AMI research. Patients Acceptance and Comprehension to Written and Verbal Consent (PAC–VC) compared patients’ understanding and attitudes to verbal and written consents in AMI RCTs.

**Methods:**

PAC–VC recruited patients from 3 AMI trials using both verbal N = 12 and written N = 6 consents. We compared patients’ understanding using two survey questionnaires. The first questionnaire used open-ended questions with multiple choice answers. The second questionnaire used a 5-point Likert scale to measure patients understanding and attitudes to the consent process. Overall answers average scores were categorized into three groups: Adequate understanding (71–100) %, Partial understanding (41–70)% and Inadequate understanding (0–40)%.

**Results:**

Responses showed patients with verbal assent had adequate understanding to most components of informed consent, close to those of written consent. Most patients did not read written information entirely and believed that it is not important to make a final decision. Patients favoured to have written information be part of the consent but not necessarily presented during the initial consent process. Patients felt less pressured in the verbal assent arm than those of written consent.

**Conclusion:**

Patients had adequate understanding to most components of verbal assent and comparable to those of written consent. Utilizing verbal assents in the acute care setting should be further assessed in larger trials.

## Background

The concept of informed consent has a legacy of going back to the 1767 when an English court prevented experimentation on patients without obtaining consent [1]. Since the inception of this concept, informed consent has gone through many adjustments to reach the current definition. The International Council of Harmonization (ICH) defines informed consent as a process by which subjects voluntarily confirm their willingness to participate in a study after having been informed of all aspects relevant to the subjects’ decision. Informed consent relies on three principles: disclosure of adequate information about the study, subjects understand the information provided, and voluntariness to give consent [2].

Randomized clinical trials (RCTs) are essential to develop new treatment strategies as well as to refine existing ones for acute and chronic medical conditions. Acute myocardial infarction (AMI) research presents a special challenge. It often requires enrollment of critically ill patients who are distressed and require urgent therapy to prevent morbidity and potential mortality. Evidence suggests that patients in this category are generally in a suboptimal state to understand or remember facts related to their condition and planned interventions [3–5]. It also suggests that many patients who consent to AMI trials remember the main information, however their degree of understanding and perceived comprehension was subjective and often questionable [6]. Patients in this category of AMI research found it irrational to be expected to read written material in an acute phase of a heart attack [6]. Instead, patients turn to oral information and explanations as a substitute [4, 7, 8].

Despite the current evidence, verbal assent has not been formally examined nor compared to the conventional written consents. Additionally, previous studies assessed patient comprehension by simply seeking their subjective opinions about their level of understanding or using the degree of information recollection as surrogate measure. Patients Acceptance and Comprehension to Written and Verbal Consent (PAC–VC) is designed to compare patient’ perspectives and understanding to verbal and written consents in AMI trials.

## Methods

### Study design

This was a descriptive, survey design, questionnaire-based study conducted at the Mazankowski Alberta Heart Institute from April 2014 to June 2015. During this time, PAC–VC interviewed patients already enrolled into 3 ongoing AMI RCTs: Remote ischemic conditioning in ST-Elevation myocardial infarction research (REMCON-STEMI), Complete versus Culprit-only Revascularization to Treat Multi-vessel Disease After Early PCI for STEMI (COMPLETE) and routine aspiration ThrOmbecTomy with PCI versus PCI ALone in patients with STEMI undergoing primary PCI (TOTAL). TOTAL and COMPLETE trials used the traditional written consent process in recruitment by providing written material to patients for review, then consent would be obtained for enrollment. on the other hand, REMCON-STEMI trial operated differently in obtaining consent. The trial consented patients using verbal script read by Emergency Medical services (EMS) personnel during enrollment to obtain a verbal assent. The process involved only reading a script to study participants in the ambulance while being transported to the hospital with no supplementary material to review for reference. Once patients are stable and received their treatment, a second formal written consent is obtained by a research nurse within 72 h of their initial enrollment with supplementary written material provided. The difference in consent process between REMCON-STEMI trial and the other two trials was the absence of any written information for patients to review. Approval from our research ethics board was obtained prior to initiating recruitment. Written information readability grade level ranged from 10 to 13 at the Flesch–Kincaid Grade Level test.

### Recruitment

**Fig. 1:**
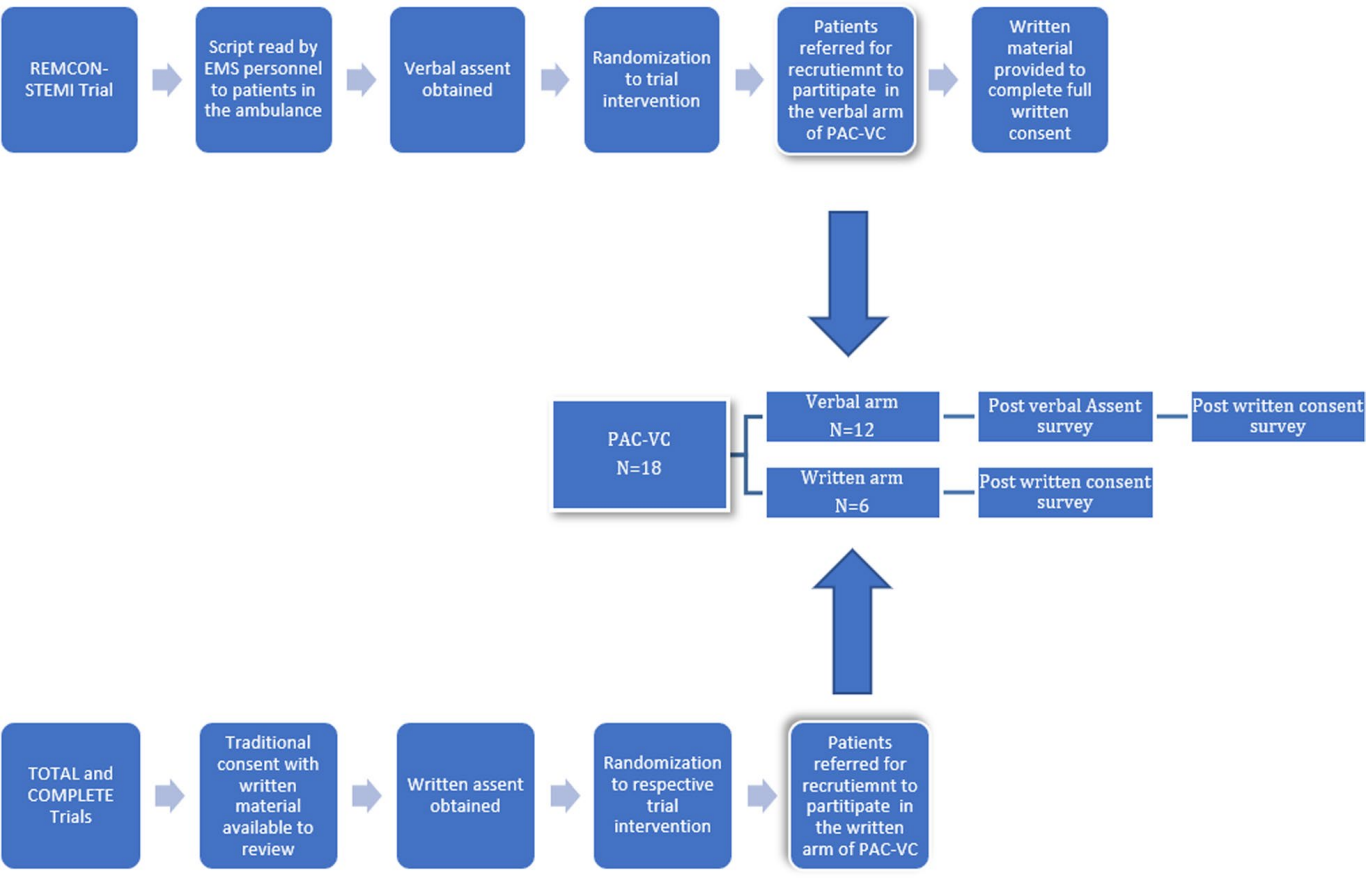
PAC–VC methods flow chart

Once patients were randomized to one of the determined 3 AMI trials, notification was sent to the research team to screen potential patients for eligibility. No formal sample size calculation was completed. Based on the principal investigator’s experience, an ideal number of subjects to be enrolled was approximately 40 patients. This number was only an estimation and was expected that it may have to be altered depending on the nature of the data obtained, and the accessibility of patients recruitment to the original AMI trials. Of 21 patients initially approached, 18 agreed/consented to participate. PAC–VC enrolled patients into two parallel arms: verbal and written. The verbal arm consisted of patients from the REMCON-STEMI trial who provided verbal assent and had not yet provided written consent. The written arm consisted of patients from the COMPLETE and TOTAL trials. Patients from all studies were approached within 72 h from their randomization to the original AMI trials once deemed medically stable. Patients were invited to participate in PAC–VC. Once agreed, assessment of their comprehension was done by administering a survey questionnaire as shown in Fig. 1.

### Study population

All patients who consented to REM-CON, COMPLETE and/or TOTAL trials were screened for eligibility. Patients were excluded if they were unconscious, hemodynamically unstable, has dementia or mental illness or patients who gave consent through a proxy or a substitute decision maker.

### Assessment tools and data collection

PAC–VC used two sets of survey questionnaires to assess qualitative and quantitative responses of the subjects. The first set used open-ended questions with multiple choice answers. The questions tested patients’ understanding of the core components of informed consent. This part was designed to objectively test patients’ understanding. The second part used a Likert scale to measure perceived patients’ understanding about the trials in addition to the assessment of patients’ attitudes and perspectives towards the consent process. Both parts of the questionnaire were reviewed by the research team in addition to an external expert to assess relevance of the content. Once patients agreed to participate in the study, and signed the consent form, questionnaires were provided in a paper format, handed to patients to review and respond to the questions at their convenience. Responses were retrieved later on the same day.

### Scoring

Patients’ responses for the objective assessment (Part 1) were assigned scores according to the answers. Correct answers were granted a complete score of 100%. Incorrect answers were scored 0%. “Do not know” responses were considered as partial awareness given the insight to lack of knowledge and granted a score of 50%. Average scores were calculated for the consent components assessed through the questionnaire. Overall scores were categorized into Adequate understanding (71–100%), Partial understanding (41–70%) and Inadequate understanding (0–40%). These cutoffs scores were chosen arbitrary as there was no previous consensus to the definition of adequacy of understanding.

Statistical Package for the Social Sciences (SPSS) was used for all analysis. Simple descriptive statistics were used to report the number of patients responses and their percentages in addition to their average scores per question per arm. Although the analysis was not intended to assess for statistically significant differences, T-Test was used to compare patients’ mean scores for any significant differences (*P* = 0.5).

### Patient characteristics

PAC–VC enrolled 18 AMI RCTs participants divided into two arms according to the initial informed consent used: 12 participants in the verbal assent arm and 6 participants in the written consent arm. Males were 83.3% of the total participants. Median age was 54 years of age. English was the first language for 72.2% of participants and fifty percent had college education. Previous history of MI was observed in only one patient. Previous enrollment into research was reported by 4 (24%) patients. Previous ambulance transport and hospital admissions were reported by 52.9% and 70.6% of participants respectively. Attention, stress, pain and anxiety rated by the participants on a scale from 1 to 10 during the consent process is shown in Table 1.

**Table 1:** Baseline characteristics (consent type)

	Verbal	Written	Total
N (%)	12 (66.7)	6 (33.3)	18
Males	11 (91.7)	4 (66.7)	15( 83.3)
Age	60.83 (Median 57.5)	48.83 (Median 51.5)	56.83 (M = 54)
1st language is english	7 (58.3)	6 (100)	13 (72.2)
College education	6 (50)	3 (50)	9 (50)
Previous history			
MI	1 (9)	0	1(6)
Research	3 (27.3)	1 (16.7)	4 (24)
Hosp. admission	9 (81.8)	3 (50)	12 (70.6)
Ambulance transport	6 (54.5)	3 (50)	9 (52.9)
Physcal symptoms (Mean out of 10)			
Attention	5.73	6.17	5.88
Stress	7.18	6.67	7
Pain	5.27	5	5.18
Anxiety	6.91	6.83	6.88

### Patients’ degree of understanding and comprehension

**Fig. 2:**
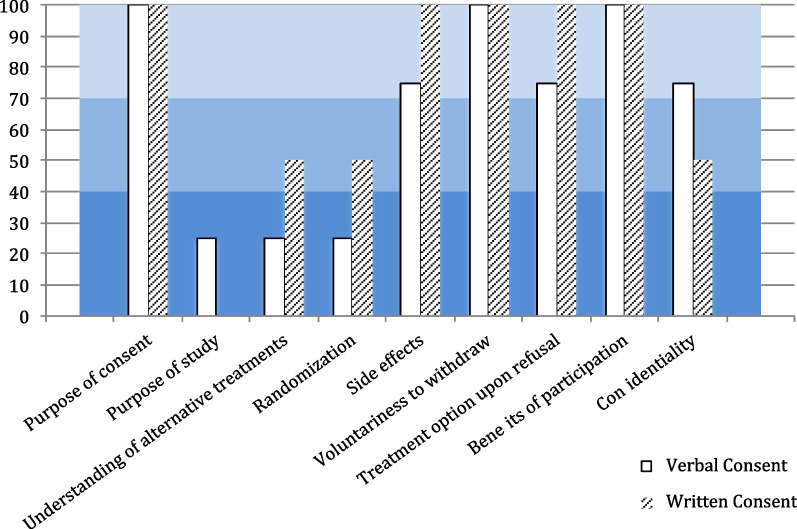
Total score of patients’ understanding to the components of consent

Responses showed that patients had adequate understanding of most core components of the verbal assent and was comparable to the understanding of written consents as shown in Fig. 2.

Participants from both arms had adequate comprehension to the purpose of consent with scores 91.7% versus 100% in the verbal and written consent groups respectively, however they had a partial understanding to the details when asked about the purpose of the study with average scores 41.67% and 66.7% in verbal and written consents respectively. This was consistent with the overall perspective of patients in both groups as demonstrated by the responses in the second questionnaire (Tables 2 and 3).

**Table 2:** Objective questionnaire scores described in means out of 100

Consent component	Consent type	
	Verbal assent	Written consent
Purpose of consent	91.67	100.00
Purpose of study	41.67	66.67
Duration of study	45.83	25.00
Nature of study intervention	50.00	66.67
Number of study groups	50.00	75.00
Understanding of alternative treatments	33.33	33.33
Randomization	37.50	50.00
Blindness	25.00	25.00
Side effects	70.83	33.33
Contacts in case of side effects	62.50	25.00
Compensation in case of harm	58.33	75.00
Voluntariness of withdraw	83.33	91.67
Treatment options if refused to participate	75.00	83.33
Benefits of participation	70.00	100.00
Financial benefits of participation	83.33	91.67
Confidentiality	83.33	83.33
Whom to contact for any complaints	83.33	75.00
Total Score	61.47	64.71

**Table 3:** Patients Subjective Understanding

Items examined	Consent type			
	Verbal assent		Written consent	
	Count	N %	Count	N %
*You understand the purpose of study*				
Agree	1	8.3	0	0.0
Cannot decide	2	16.7	0	0.0
Disagree	9	75.0	6	100.0
*You know how long you will be enrolled in this study*				
Agree	3	25.0	3	50.0
Cannot decide	4	33.3	0	0.0
Disagree	5	41.7	3	50.0
*You understand what will be done in this study and what you are being asked to do*				
Agree	1	8.3	1	20.0
Cannot decide	3	25.0	0	0.0
Disagree	8	66.7	4	80.0
*You recognize the experimental part that may be used in your treatment. (Study intervention)*				
Agree	1	8.3	0	0.0
Cannot decide	2	16.7	0	0.0
Disagree	9	75.0	6	100.0
*You recognize the possible risks or discomforts that may result due to participation in this study*				
Agree	0	0.0	0	0.0
Cannot decide	2	16.7	1	16.7
Disagree	10	83.3	5	83.3
*You recognize the possible benefits you may gain from participation*				
Agree	0	0.0	0	0.0
Cannot decide	2	16.7	0	0.0
Disagree	10	83.3	6	100.0
*You recognize the possible benefits that may help future patients*				
Agree	0	0.0	0	0.0
Cannot decide	0	0.0	0	0.0
Disagree	12	100.0	6	100.0
*You know alternative options/treatments you may have if you had chosen to ****NOT**** participate*				
Agree	2	16.7	1	16.7
Cannot decide	1	8.3	1	16.7
Disagree	9	75.0	4	66.7
*You understand that your information is being kept confidential and disclosed only to authorized personnel*				
Agree	0	0.0	0	0.0
Cannot decide	0	0.0	0	0.0
Disagree	12	100.0	6	100.0
*You know whom you should contact in case of side effects or injuries that may result due to participation in the study*				
Agree	1	8.3	2	33.3
Cannot decide	2	16.7	0	0.0
Disagree	9	75.0	4	66.7
*You know what compensation or treatment is available for you in case of side effects or injury*				
Agree	3	25.0	3	50.0
Cannot decide	3	25.0	0	0.0
Disagree	6	50.0	3	50.0
*You understand that your participation is completely voluntary and it is not going to affect your treatment if you choose to withdraw*				
Agree	0	0.0	0	0.0
Cannot decide	0	0.0	0	0.0
Disagree	12	100.0	6	100.0
*You understand that you can withdraw from this study at any time you wish to do so*				
Agree	0	0.0	0	0.0
Cannot decide	0	0.0	0	0.0
Disagree	12	100.0	6	100.0
*You know whom you should contact in case you have questions, comments, concerns or complaints about the study*				
Agree	2	16.7	2	33.3
Cannot decide	2	16.7	0	0.0
Disagree	8	66.7	4	66.7

The concept of randomization was challenging to participants in both groups where they showed inadequate to partial comprehension with an average score of 37.5% and 50% in the verbal and written consents respectively. The difference in scores did not reach statistical significance. Participants’ comprehension to the risks was adequate in the verbal group while inadequate in the written group 70.8% versus 33.3%. On the other hand, both groups showed adequate comprehension to the benefits with average scores 70% and 100% in the verbal and written consent arms respectively.

Study participants showed adequate comprehension to the concept of autonomy and treatment alternatives in both arms with an average score of 83.3% versus 91.7% and 75% versus 83.3% in the verbal and written groups respectively.

Participants in both groups equally showed an adequate comprehension to confidentiality with an average score of 83.3% in both groups.

### Patients perspectives and attitudes

Only 33.3% of patients read the written information. Most patients, 75% of the verbal arm and 100% of the written arm did not think written information was very important in making the final decision to choose to participate or not. However, participants from the verbal assent (75%) still wanted to have written information to be part of the consent process and only 25% of verbal and 16.7% of written arms wanted the written information to be presented during the initial consent process. Majority of patients in the written consent group (83.3%) and 50% of the verbal assent group felt pressured during the consent process. Also, 75% of patients in the verbal assent arm, and 100% of the written consent arm felt that the consent process was not satisfactory. (Table 4).

**Table 4:** Patients perspectives

	Consent type	
	Verbal assent	Written consent
	N %	N %
*I would prefer only verbal information presented during the consent process*		
Agree	2 (16.7%)	2 (33.3%)
Cannot decide	1 (8.3%)	2 (33.3%)
Disagree	9 (75.0%)	2 (33.3%)
*I would prefer written information presented during the consent process*		
Agree	3 (25.0%)	1 (16.7%)
Cannot decide	3 (25.0%)	3 (50.0%)
Disagree	6 (50.0%)	2 (33.3%)
*I read the written information about the research study before making my decision*		
Agree	2 (16.7%)	2 (33.3%)
Cannot decide	2 (16.7%)	0 (0.0%)
Disagree	8 (66.7%)	4 (66.7%)
*I believe written information is very important in making my final decision to participate*		
Agree	2 (16.7%)	0 (0.0%)
Cannot decide	1 (8.3%)	0 (0.0%)
Disagree	9 (75.0%)	6 (100.0%)
*I feel satisfied and comfortable with the consent process*		
Agree	0 (0.0%)	0 (0.0%)
Cannot decide	3 (25.0%)	0 (0.0%)
Disagree	9 (75.0%)	6 (100.0%)
*I felt pressured by time when I made my decision during the consent process*		
Agree	6 (50.0%)	5 (83.3%)
Cannot decide	3 (25.0%)	1 (16.7%)
Disagree	3 (25.0%)	0 (0.0%)

### Post-verbal/post-written consent interviews

**Fig. 3:**
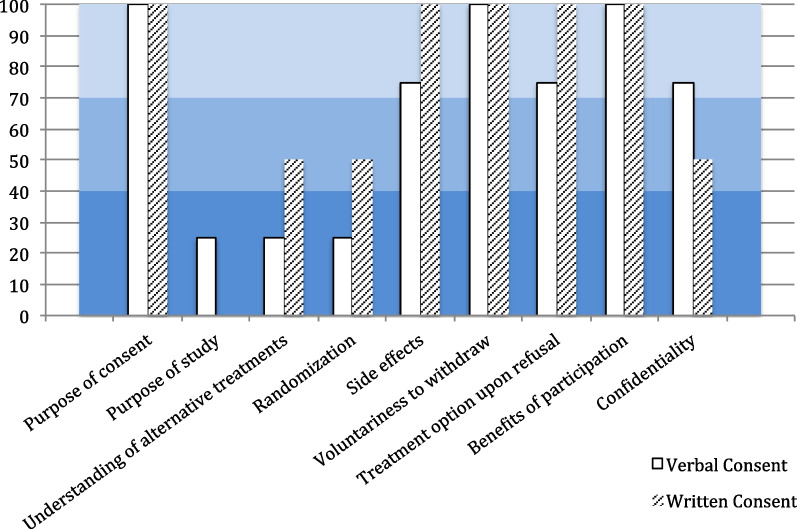
Post-verbal/post-written consent interviews responses

REMCON-STEMI patients in the verbal arm were asked to take part of the questionnaire for a second time once completed the formal written consent. Among 12 patients, only 2 agreed to answer the questionnaire for the 2nd time. Overall responses showed knowledge improvement in some areas as displayed in Fig. 3. Interestingly, patients’ attitudes and opinions did not change after exposure to the written consent.

## Discussion

The role of AMI clinical trials in developing and refining treatment guidelines is essential. However, the emergency nature of AMI as a disease poses extra challenges to obtain an ideal informed consent.

To our knowledge, PAC–VC is the first study to utilize a questionnaire that objectively compares patients’ perspectives and comprehension of verbal assent to written consent. Our results show that patients understanding of verbal assent is comparable to written consents with an adequate understanding of most core components of the consent information. These include the purpose of consent, idea of autonomy, benefits, alternative treatments, choice to refuse participation and confidentiality. However, when attention to details was required, participants showed partial to inadequate understanding. Examples include the concept of randomization, blinding, alternative treatments and side effects. These results confirm previous findings demonstrating that patients who consent to clinical trials remember general information about studies, however often have sub-optimal understanding of the specific study details [4, 7, 9–13]. This was clearly illustrated in our cohort when participants performed poorly in understanding alternative treatments, randomization and side effects. Abstract thinking and complex processing is required to interpret these components and may not be possible and difficult to handle by severely ill patients in an acute phase of a disease. Additionally, it has also been previously suggested that poor understanding and recall to side effects may be influenced by patients inability to accept potentially unpleasant realities [14]. Interestingly, patients in the verbal arm of our cohort showed an adequate understanding of treatment side effects which may argue that verbal information is easier to understand and emphasize the importance of this tool of information delivery.

Most patients in our cohort did not read the provided written information and reported that they did not believe it was very important in making their final decision in regards to participation. Patients did support the importance of having written information available, yet not necessarily to be presented during the acute phase of the consent process. These findings are consistent with the literature as was found that patients did not read the written material provided to them prior to making decisions [4, 8, 11–13, 15]. Instead, patients preferred a summary of verbal information and turned to oral explanations as a substitute. Interestingly, 75% of patients in the verbal assent arm, and 100% of the written consent arm felt that the consent process was not satisfactory. Such impressions require further exploration.

Delay in treatment of AMI increases the rate of adverse outcomes and risk of death [16–19]. Hence, the consent process required for participation in AMI trials might unduly pressure potential participants and influence patients willingness to consent. The majority of patients (83.3%) in the written consent arm felt pressured during the consent process. It has been previously shown that participants felt pressured at the time of consent process and rushed into making a prompt decision, which put participants under stress [11, 15]. These findings were less observed in the verbal assent arm (50% of participants). This can be interpreted as reading needs more time and special attention to analyze the facts enlisted in a written format, on the other hand, patients may find oral information and explanations as an easier substitute to process the data and make a quicker decision without feeling pressured.

These findings come in support to advocate for more utilization of verbal assents to enroll patients in research that involves vulnerable, sick patients. These patients are usually met in stroke, trauma, and acute myocardial infarction research where they can experience symptoms that may render their abilities to read, process and understand written information. Patients mostly make decisions and consent to enroll before reading the written information [4, 8, 11–13, 15]. Recognizing this fact makes importance of verbal assent as a method of information delivery greater. This in theory can improve the quality of the consent process and potentially save a much-needed time to implement study interventions and expedite patients care. Such methods of consent and information provision can have their own challenges. Administration of such information in a vernal form needs special recruiters training, to ensure consistency, and quality in information delivery. Safeguards can be added throughout the recruitment process to ensure patients understanding. These can include frequent questions to engage patients in a discussion and clarify inquiries about study.

## Strengths and limitations

This study to our knowledge is the first to assess patients understanding of verbal assents in comparison to written consents in AMI research. PAC–VC used a multiple-choice questionnaire to objectively assess patients understanding in contrast to previous studies using self-reporting. Unfortunately, there are no standardized testing to quantify comprehension or define how much understanding is satisfactory. Since there are no grading systems, we opted to construct our own to simplify the interpretations of the results. This is certainly a point that can be debated, and may set a stage for further discussions to clarify the definitions of adequate and poor understanding. These are still subjective and one can argue that optimal understanding would require additional set of measures and standards. Study participants were recruited from different AMI trials addressing multiple research questions with diverse complexity and study protocols. Although the COMPLETE trial recruited patients with AMI, patients were in a stable condition in contrary to the acute patients recruited to REMCON and TOTAL. Finally, the study is small, non-randomized with heterogenous population characteristics (gender, age, education levels) that would plead to cautiously interpret the results to propose a question and an idea rather than claim conclusions.

## Conclusion

PAC–VC is a small, non-randomized prospective study assessing patients’ comprehension to verbal assent and written consent in AMI research. Patients had adequate understanding to most components of verbal assent and comparable to those of written consent. Although patients still prefer written information to be part of the consent process, most do not read the written information nor feel that it should necessarily be presented during the acute phase of the consent process. The study results suggest that verbal assent may have a future role as acceptable alternative to the traditional written consent in similar AMI research. These results invite more research in this area with larger studies.

## Data Availability

The datasets used and/or analysed during the current study available from the corresponding author on reasonable request.

## Abbreviations

PAC–VC: Patients acceptance and comprehension to written and verbal consent
AMI: Acute myocardial infarction
RCT: Randomized clinical trials
ICH: The International Council of Harmonization
REMCON-STEMI: Remote ischemic conditioning in ST-elevation myocardial infarction research
COMPLETE: Complete versus culprit-only revascularization to treat multi-vessel disease after early PCI for STEMI
TOTAL: Routine aspiration ThrOmbecTomy with PCI versus PCI alone in patients with STEMI undergoing primary PCI
EMS: Emergency medical services
